# Factors Attenuating Zinc Deficiency Improvement in Direct-Acting Antiviral Agent-Treated Chronic Hepatitis C Virus Infection

**DOI:** 10.3390/nu10111620

**Published:** 2018-11-02

**Authors:** Yi-Ling Ko, Daisuke Morihara, Kumiko Shibata, Ryo Yamauchi, Hiromi Fukuda, Hideo Kunimoto, Kazuhide Takata, Takashi Tanaka, Shinjiro Inomata, Keiji Yokoyama, Yasuaki Takeyama, Satoshi Shakado, Shotaro Sakisaka

**Affiliations:** Department of Gastroenterology, Fukuoka University Faculty of Medicine, 7-45-1 Nanakuma, Jonan-ku, Fukuoka 814-0180, Japan; withoutatracemichelle@gmail.com (Y.-L.K.); kumi.k0402935@gmail.com (K.S.); md170029@cis.fukuoka-u.ac.jp (R.Y.); hfukuda@fukuoka-u.ac.jp (H.F.); hideokunimoto@yahoo.co.jp (H.K.); edihuzak_t@yahoo.co.jp (K.T.); tanaka329@yahoo.co.jp (T.T.); sinomata@fukuoka-u.ac.jp (S.I.); yokotin@fukuoka-u.ac.jp (K.Y.); yaz@fukuoka-u.ac.jp (Y.T.); shakado@cis.fukuoka-u.ac.jp (S.Sh.); sakisaka@fukuoka-u.ac.jp (S.Sa.)

**Keywords:** zinc deficiency, hepatitis C virus, sustained virologic response, direct-acting antiviral agent, hypozincemia, hyperuricemia

## Abstract

Zinc deficiency is frequently observed in chronic liver diseases. However, no studies have focused on the zinc status in chronic hepatitis C (HCV)-infected patients receiving direct-acting antiviral agents (DAAs). In this retrospective study, we assessed the serum zinc status in DAA-treated HCV patients with sustained virologic response for over two years (Zn-2y). Ninety-five patients were enrolled, whose baseline characteristics and blood parameters at DAA therapy initiation were collected. Baseline Zn < 65 µg/dL (odds ratio (OR) = 10.56, *p* < 0.001) and baseline uric acid (UA) > 5.5 mg/dL (OR = 9.99, *p* = 0.001) were independent risk factors for Zn-2y deficiency. A decision-tree algorithm classified low-baseline Zn and high-baseline UA as the first two variables, suggesting that baseline hypozincemia and hyperuricemia are prognosticators for long-term zinc deficiency. Baseline Zn was negatively correlated with the Fibrosis-4 (FIB-4) index, while baseline UA was significantly higher in habitual alcohol drinkers. In conclusion, serum zinc levels should be closely monitored, considering that zinc status improvement is related to liver fibrosis regression. Hyperuricemia indicates risks of developing metabolic disorders and subsequent zinc deficiency, for which an adjustment of personal lifestyle or dietary habits should be recommended clinically.

## 1. Introduction

Zinc is an essential trace element, whose biological functions can be divided into three categories: catalytic activity of enzymes, structural integrity of proteins, and regulation of gene expression [1]. Approximately 250 proteins contain zinc, including enzymes such as angiotensin-converting enzyme, alkaline phosphatase, deoxyribonucleic acid (DNA) and ribonucleic acid (RNA) polymerase, copper–zinc superoxide dismutase, and metallothionein. Zinc absorption occurs mainly in the small intestine. Approximately 60% of the total body zinc is found in bone/muscle pools, and has a slow turnover [2]. In the blood, zinc circulates at a concentration of 70 µg/dL to 120 µg/dL with 60% loosely bound to albumin and 30% tightly bound to macroglobulin [3]. 

Zinc possesses anti-inflammatory, anti-fibrogenic, and anti-carcinogenic effects in various liver diseases [4]. Zinc supplementation exerts an anti-inflammatory effect on the liver in chronic hepatitis C virus (HCV)-infected patients by reducing iron overload [5]. Moreover, several studies have revealed enhanced responses or tolerance to interferon therapy by zinc administration to HCV infected patients [6,7,8,9]. However, zinc deficiency is also frequently observed in chronic liver diseases. In a Japanese multiple-institute study, the mean blood zinc level was 64.1 μg/dL in patients with liver cirrhosis [10]. In addition, the zinc serum level significantly decreased, which tends to negatively correlate with hepatic functional reserve, in patients with both alcohol and HCV-related liver diseases [11].

However, to the best of our knowledge, no studies have focused on the zinc status of HCV patients who received direct-acting antiviral agent (DAA) therapy. Thus, we have assessed the change in serum zinc levels and its association with other blood parameters in DAA-treated HCV patients who have achieved sustained virologic response (SVR) for over two years. We shall present recommendations on the surveillance and treatment of zinc deficiency for clinical practice based on our results.

## 2. Materials and Methods

### 2.1. Patients

Two hundred and fifty-one patients were treated with DAAs for chronic HCV infection from September 2014 to March 2016 in Fukuoka University Hospital. In this retrospective study, 128 patients, who had continuous follow-up for over two years after achieving SVR, were enrolled. According to our clinical records, none of the patients received supplementation of zinc, iron, or calcium during the observation period. No excessive caffeine intake was noted.

### 2.2. Variables for Analysis

Thirty-three patients were excluded due to insufficient laboratory data. We collected the patient history and laboratory data of the remaining 95 subjects by checking their electronic clinical records. The serum zinc level at the time of DAA therapy initiation was defined as the baseline zinc (baseline Zn) level, while the zinc level measured two years after achieving SVR was defined as the two-year zinc (Zn-2y) level. In addition to these zinc levels, patient characteristics such as sex, age, body mass index (BMI), alcohol consumption, and DAA regimens were recorded. Habitual alcohol drinking was defined as ethanol consumption of more than 60 g per day [12].

The laboratory data shown in Table 1 were analyzed when DAA therapy was initiated. Body mass index (BMI) was calculated as the weight in kilograms divided by height in meters squared. The Fibrosis-4 (FIB-4) index was calculated using the following formula [13]:

FIB-4 index = Age (years) × AST (U/L)/(Plt (10^9/^L) × ALT^1/2^ (U/L))

where AST is aspartate aminotransferase, Plt is platelets, and ALT is alanine aminotransferase.

### 2.3. Statistical Analysis

Zinc deficiency was defined as a serum zinc level < 70 µg/dL [3,10]. We divided the patients into two groups by zinc level, <70 µg/dL and ≥70 µg/dL. We used JMP version 13.0 software (SAS Institute, Charlotte, NC, USA) for statistical analysis. The baseline characteristics and all of the blood parameters mentioned above, including baseline Zn levels, were analyzed and compared between the Zn-2y < 70 µg/dL and Zn-2y ≥ 70 µg/dL groups by Chi-square tests and multiple regression analyses, to determine the risk factors of zinc deficiency. Since this is a retrospective observational study, the numbers of patients enrolled were limited by the existing data and preserved blood specimens. As a result, we consulted the professional statisticians in our institute concerning the relatively small sample size, and they recommended stepwise multiple regression for data analysis.

### 2.4. Ethics

This study protocol conformed to the ethical guidelines of the 1975 Declaration of Helsinki and was conducted under approval given by the institutional review board of Fukuoka University.

## 3. Results

### 3.1. Overall Characteristics

Ninety-five chronic HCV infected patients receiving DAA therapy from September 2014 to March 2016 were enrolled in this study. The DAA regimens were asunaprevir/daclatasvir (ASV/DCV, 50 patients, 52.6%), sofosbuvir/ledipasvir (SOF/LDV, 41 patients, 43.2%), and ombitasvir/paritaprevir/ribavirin (OBV/PTV/r, four patients, 4.2%). The patient characteristics are shown in Table 1. There were 44 males (46.3%), and the mean age was 68 years. Zinc deficiency was present in 46 of 95 patients (48.4%) at the start of DAA therapy, and 16 of 95 patients (16.8%) two years after SVR. The mean value of the FIB-4 index was 5.195, and the mean AST and ALT levels were 52.6 U/L and 50.1 U/L, respectively. Twenty patients (21.1%) were habitual alcohol drinkers, while the remainder either drank alcohol socially or abstained. 

### 3.2. Risk Factors of Zinc Deficiency Two Years after SVR

As shown in Table 2, multiple regression analysis revealed that a serum zinc level under 65 µg/dL (baseline Zn < 65 μg/dL; odds ratio (OR) = 10.56; 95% confidence interval (CI), 3.00–45.27; *p* < 0.001) and a uric acid (UA) level over 5.5 mg/dL (baseline UA > 5.5 mg/dL; OR = 9.99; 95% CI, 2.31–71.31; *p* = 0.001) were independent risk factors of zinc deficiency two years after SVR (Zn-2y < 70 µg/dL). 

**Figure 1:**
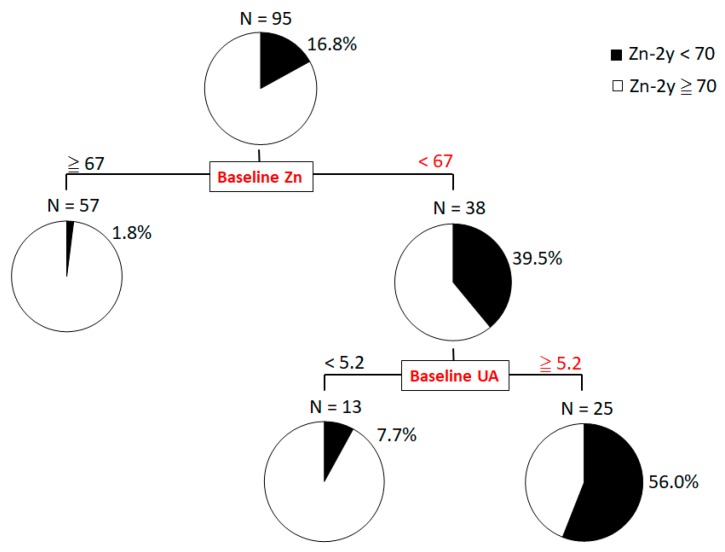
Decision-Tree Algorithm for Zn-2y Deficiency. The decision-tree algorithm for specifying the factors leading to Zn-2y < 70 µg/dL, showed that low baseline Zn (Zn < 67 μg/dL) was the first divergence variable, and high baseline UA (UA > 5.2 mg/dL) was the variable for second classification. For patients with both baseline Zn < 67 μg/dL and baseline UA > 5.2 mg/dL, 56.0% presented with Zn-2y < 70 µg/dL. UA, uric acid; Zn-2y, zinc level two years after sustained virologic response.

A decision-tree algorithm (Figure 1) was created to specify the factors leading to Zn-2y < 70 µg/dL. Baseline Zn < 67 μg/dL was the first divergence variable, and baseline UA > 5.2 mg/dL was the second classification variable. The result was the same as that from the multiple regression analysis, which confirms that low baseline Zn and high baseline UA are factors with significant impact on Zn-2y deficiency. Among the patients with baseline Zn < 67 μg/dL, 39.5% had Zn-2y < 70 µg/dL. Moreover, for patients with both baseline Zn < 67 μg/dL and baseline UA > 5.2 mg/dL, 56.0% had Zn-2y < 70 µg/dL.

### 3.3. Factors Related to Baseline Zn and Baseline UA

**Figure 2:**
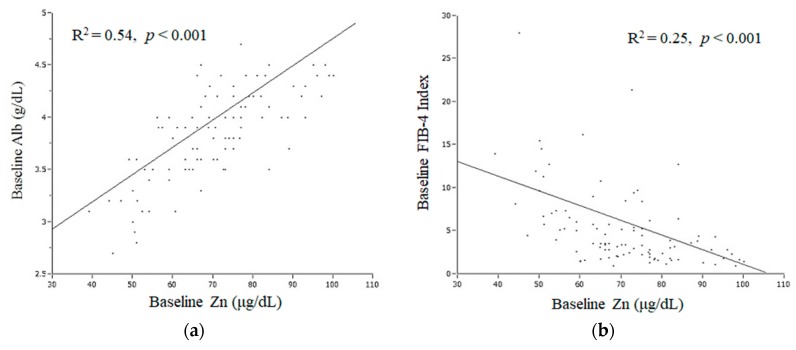

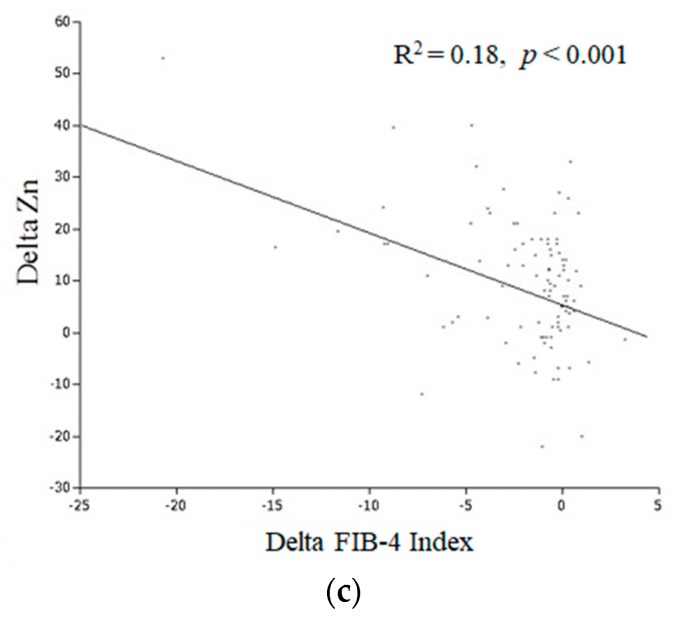
Relationship between Baseline Zn and Other Parameters. (**a**) Positive correlation (*R*^2^ = 0.54, *p* < 0.001, linear regression analysis) between baseline Zn and baseline Alb level. (**b**) Negative correlation (*R*^2^ = 0.25, *p* < 0.001, linear regression analysis) between baseline Zn and baseline FIB-4 index. (**c**) Change in serum zinc level (delta Zn) and change in FIB-4 index (delta FIB-4 index) from initiation of DAA therapy to two years after achieving SVR showed a negative correlation (*R*^2^ = 0.18, *p* < 0.001, linear regression analysis). Alb, albumin; DAA, direct-acting antiviral agent; SVR, sustained virologic response.

A positive correlation (*R*^2^ = 0.54, *p* < 0.001) between baseline Zn and serum albumin level (baseline Alb), and a negative correlation (*R*^2^ = 0.25, *p* < 0.001) between baseline Zn and the FIB-4 index at the initiation of DAA therapy (baseline FIB-4 index), were identified using linear regression analysis (Figure 2a,b). In addition, the change in serum zinc level (delta Zn) and change in FIB-4 index (delta FIB-4 index) from the initiation of DAA therapy until two years after achieving SVR showed a negative correlation (*R*^2^ = 0.18, *p* < 0.001) by linear regression analysis (Figure 2c).

**Figure 3:**
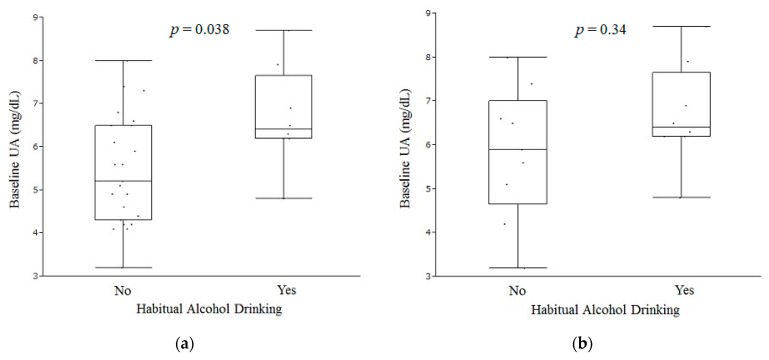

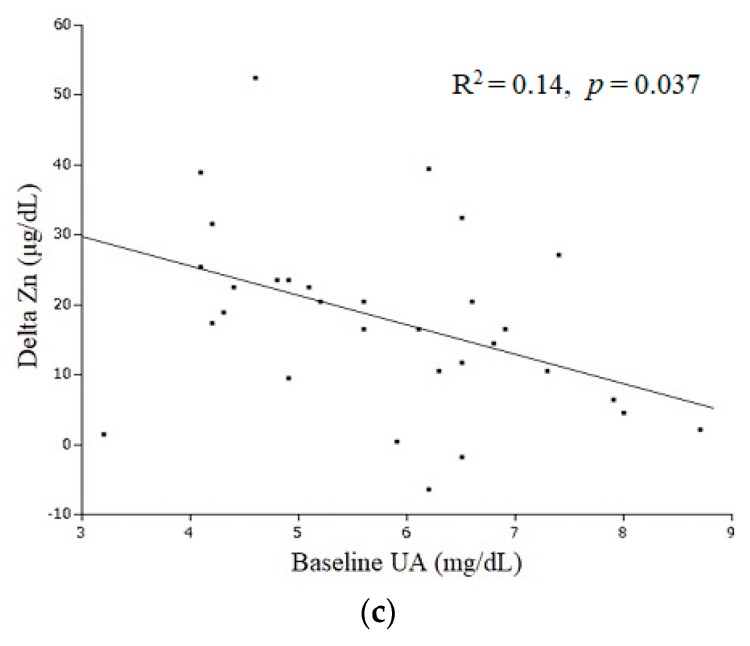
Relationship between Baseline UA and Other Parameters. (**a**) In the baseline Zn < 65 μg/dL (31/95, 32.6%) subgroup, patients having habitual alcohol drinking (8/31, 25.8%), showed significantly higher baseline UA levels (mean UA, 6.69 vs. 5.46, *p* = 0.038, Wilcoxon test). (**b**) Baseline UA levels in male patients with habitual alcohol drinking (8/17, 47.1%) showed the same tendency of higher baseline UA as in part (**a**). However, no significant difference was found. (**c**) In the baseline Zn < 65 μg/dL (31/95, 32.6%) subgroup, baseline UA levels were negatively correlated with delta Zn (*R*^2^ = 0.14, *p* = 0.037, linear regression analysis). UA, uric acid.

In the baseline Zn < 65 μg/dL (31/95, 32.6%) subgroup, habitual alcohol drinkers (8/31, 25.8%), had a significantly higher baseline UA level than the other patients did (mean UA, 6.69 versus 5.46, *p* = 0.038, Wilcoxon test) (Figure 3a). The baseline UA level in male habitual alcohol drinkers (8/17, 47.1%), despite a tendency toward higher baseline UA, showed no significant difference compared with the baseline UA level of those who were not habitual alcohol drinkers (Figure 3b). In the baseline Zn < 65 μg/dL subgroup, the baseline UA level had a negative correlation with delta Zn (*R*^2^ = 0.14, *p* = 0.037, linear regression analysis) (Figure 3c). 

## 4. Discussion

To the best of our knowledge, this is the first study to clarify the risk factors of long-term zinc deficiency in chronic HCV-infected patients receiving DAA therapy. The most valuable and novel finding of this study is that baseline hypozincemia and hyperuricemia are independent risk factors of persistent zinc deficiency in these patients, despite SVR achievement. Previously, a slight and non-significant decrease in serum zinc levels was reported in HCV patients during interferon therapy, compared with their zinc levels before and after therapy [14]. Greater decreases in serum zinc after interferon administration tended to occur in patients with higher levels of basal zinc, which may have resulted from a flux of serum zinc to the liver created by interferon-induced hepatic metallothionein synthesis for anti-viral effects [9,15]. In addition, the serum zinc levels of HCV complete responders were higher than those of non-responders before and during interferon therapy, indicating that we may judge the therapeutic efficacy of interferon from zinc status [9]. Mean serum zinc concentrations increased during the follow-up to the greatest extent in interferon therapy responders [14]. Although the data are not shown here, no significant change (ANOVA, *p* = 0.662) in serum zinc levels before treatment initiation (78.6 μg/dL) and after SVR (75.2 μg/dL) was noted in HCV patients under interferon therapy. In the present study, 46 of 95 patients (48.4%) presented with zinc deficiency at the initiation of DAA therapy. The mean level of baseline Zn was 70.3 μg/dL, while that of Zn-2y was 80.0 μg/dL, representing a significant increase in serum zinc levels after DAA therapy (ANOVA, *p* < 0.001). However, 16 of 95 patients (16.8%) still suffered from zinc deficiency after treatment.

An imbalance between cellular and humoral immunity due to an imbalance between the Th1 and Th2 cytokines was noted in HCV infection. Furthermore, an insufficient T-cell immune response can be considered the main cause of the chronification of HCV infection. A defective antiviral immune response causes continued activation of proinflammatory cytokine secretion [16], which may subsequently alter the levels of serum trace elements [17]. For instance, interleukin 6 (IL-6), a proinflammatory cytokine that regulates the Zip14 zinc transporter in the liver, contributes to hypozincemia in the acute-phase reaction. However, the presence of oxidative stress, which is a direct consequence of mitochondria destruction by HCV, can also disrupt zinc homeostasis, since zinc acts as a signal molecule and second messenger in the redox process [16].

Baseline Zn was negatively related to the baseline FIB-4 index, which is consistent with previous findings that serum zinc levels were significantly correlated with the degree of liver fibrosis, and that serum zinc levels were significantly lower as liver fibrosis progressed [18]. In chronic liver diseases, both serum and hepatic zinc levels decrease, promoting liver fibrosis [7,19]. Zinc depletion was found to lead to collagen synthesis in a rat model [19]. In contrast, zinc administration significantly inhibited the proliferation and collagen synthesis ability of human hepatic stellate cells (HSCs) via increasing matrix metalloproteinases 13 (MMP-13) expression, which accounts for matrix degradation. The transforming growth factor beta (TGF-β) signaling pathway, which is the most powerful fibrosis-promoting cytokine in HSCs, played a role in the process. Although the specific molecular mechanism has not been elucidated, zinc might function by inhibiting TGF-β receptor 1 (TGFBR1) gene expression or by increasing TGFBR1 protein degradation [20]. Similar studies also revealed that zinc inhibits ethanol and the acetaldehyde-induced activation of HSCs, and acts as an antioxidant against the production of reactive oxygen species (ROS) by HSCs. The downregulation of tissue inhibitor of metalloproteinase (TIMP) production by zinc was also noted [21]. Likewise, in human studies, zinc supplementation decreased hepatic fibrosis, with reductions in serum markers of hepatic fibrosis, such as type-IV collagen and TIMPs, which inhibits the apoptosis of activated HSCs by the inhibition of MMP activity [19]. Other studies also indicated that liver disease was improved by zinc supplementation therapy, as serum aminotransferase levels and iron overload were reduced [5,7].

Decreased zinc levels may also be the result of liver fibrosis, which involves different mechanisms. In the blood, approximately two-thirds of circulating zinc binds to albumin. Liver fibrosis leads to hypoalbuminemia, and consequently, serum zinc concentrations decrease. Moreover, inflammation and stress, which is often associated with hypoalbuminemia, may also cause a decrease in serum zinc levels [11]. In the present study, baseline Zn was positively correlated with baseline Alb, which is consistent with the above-mentioned details. Besides hypoalbuminemia, liver fibrosis causes portal hypertension, which results in the impairment of the intestinal mucosa and formation of a portosystemic shunt. Serum zinc levels decrease as a result of decreased zinc absorption from the impaired intestinal mucosa, and they decrease further because of increased urinary zinc excretion through the portosystemic shunt [22]. Liver fibrosis may eventually progress to liver cirrhosis, when poor appetite and decreased intake may deteriorate zinc deficiency. 

We also revealed that the change in serum zinc levels was negatively associated with the change in the FIB-4 index, demonstrating that the increased zinc level was positively related to the extent of liver fibrosis improvement. Collectively, a lower baseline Zn level indicates worse liver fibrosis at baseline. Despite the clearance of the HCV, the improvement in serum zinc levels is still dependent on the liver fibrosis status.

The other independent risk factor for Zn-2y deficiency is a high baseline UA level, which was found to be significantly higher in habitual alcohol drinkers within the low-baseline Zn subgroup. In male patients, the baseline UA of those acknowledging habitual alcohol drinking showed a tendency toward a higher value, although the increase was not statistically significant, which was possibly due to the small sample size. It was previously reported that dietary zinc intake was inversely associated with hyperuricemia in a male population in China [23]. Another similar study suggested that low dietary zinc intake was significantly associated with hyperuricemia in both males and females in the United States [24]. Foods containing high concentrations of zinc, such as whole grains, dairy products, nuts, and soy products, were reported to have a protective effect against hyperuricemia [24]. It was also revealed that alcohol consumption can result in both hyperuricemia and low zinc status, which can arise from impaired intestinal absorption and increased urinary excretion [11]. 

Additionally, baseline UA was associated with delta Zn, in which increasing baseline UA was associated with reduced serum zinc level improvement. However, delta Zn showed no significant difference between patients with and without habitual alcohol drinking, which may be explained by alcohol consumption not being the only cause of both hyperuricemia and hypozincemia. Moreover, uric acid subsequently contributes to a metabolic status that is different from that generated by alcohol consumption alone (i.e., there are different prevalence rates of metabolic syndrome [25] or cardioprotective effects [26], resulting from differences in timing or the amount of alcohol ingested). Alternatively, both human studies and animal models suggest that hyperuricemia plays a role in promoting hypertension and cardiovascular disease, adipogenesis and lipogenesis, insulin and glucose dysregulation, and liver diseases [27], all of which are metabolic disorders associated with oxidative stress. Moreover, a higher serum uric acid concentration was shown to be previously associated with a higher incidence of hypertension, even in patients with a normal range of serum uric acid levels [28]. Although the data are not shown in this study, a positive correlation (correlation coefficient (*r*) = 0.43) between baseline UA and urine level of 8-hydroxy-2’-deoxyguanosine (8OHdG) after SVR achievement was revealed in 16 HCV-infected patients, suggesting that baseline UA reflects long-term oxidative status.

Regarding the relationship between zinc and uric acid-associated oxidative stress, previous studies have demonstrated that serum zinc levels decreased due to increased oxidative stress in HCV-infected patients with hepatic steatosis [29], and that liver steatosis and its severity were independently associated with hyperuricemia [30]. Accordingly, we postulate that the oxidative stress caused by metabolic problems, especially those related to uric acid, can lead to zinc consumption, since zinc is known to be an antioxidant. Zinc can attenuate oxidative stress through the introduction of metallothionein and multiple other mechanisms, such as inhibiting tumor necrosis factor and modulating multiple enzymes [11]. In addition, zinc improved the efficacy of uric acid reduction by suppressing xanthine oxidase/xanthine dehydrogenase (XOD/XDH) in a hyperuricemia mouse model [31]. Overall, a high uric acid level causes metabolic problems and results in oxidative stress, indicating that zinc may be consumed as an antioxidant and attenuate improvements in zinc levels. Different aspects of the metabolic changes caused by alcohol consumption and hyperuricemia should be further investigated.

However, overall baseline UA was not significantly related to delta Zn. This may be explained by the different impacts of baseline Zn and baseline UA. According to our decision-tree algorithm, baseline UA was the second risk factor after baseline Zn. As mentioned above, lower baseline Zn indicates more severe liver fibrosis, while higher baseline UA implies inherent risks of developing metabolic problems. These findings suggest that liver fibrosis status is the foremost risk factor affecting overall zinc improvement in patients with chronic HCV infection. Thereafter, baseline UA, which reflects increasing metabolic problems, is the second risk factor in patients with low zinc status.

## 5. Conclusions

Patients with chronic HCV infection who were treated with DAA therapy showed overall improvements in zinc deficiency over two years after SVR. Among the patients without zinc improvement, baseline hypozincemia and hyperuricemia were the prognosticators of persistent zinc deficiency, despite SVR. Hypozincemia was significantly related to hypoalbuminemia and the liver fibrotic index, indicating that zinc supplementation, even after SVR achievement, should be considered, particularly in patients with poor liver fibrotic status. The serum zinc level should also be closely monitored, considering that the improvement in zinc status is highly related to liver fibrosis regression. Moreover, in those presenting with low zinc status at baseline, hyperuricemia is significantly associated with habitual alcohol drinking, which indicates potential risks of developing metabolic disorders and subsequent zinc deficiency regardless of HCV clearance. In these cases, an adjustment of personal lifestyle or dietary habits should be strongly recommended in clinical practice.

There are some limitations in the present study. First, this was a retrospective study, and serum markers of oxidative stress could not be detected in preserved blood specimens. Future studies are required to clarify the mechanism of how zinc levels respond to uric acid-induced oxidative stress. Second, this study was conducted at a single medical institute. A larger study population from multiple centers or different races is needed. 

## Figures and Tables

**Table 1:** Patient Characteristics.

Characteristics	Values ^a^
Total Number of Patients	95
Male, No. (%)	44 (46.3)
Habitual Alcohol Drinking, No. (%)	20 (21.1)
Age, years	68.4 (9.8)
BMI, kg/m^2^	23.0 (3.5)
FIB-4 Index	5.195 (4.588)
Baseline Zn, μg/dL	70.3 (13.9)
HCV RNA, LogIU/mL	6.0 (0.6)
WBC,/μL	4251.6 (1606.3)
Hb, g/dL	13.6 (1.5)
Plt, × 10^3^/μL	140 (62)
Alb, g/dL	3.84 (0.44)
Tbil, mg/dL	1.04 (0.51)
INR	1.10 (0.08)
AST, U/L	52.6 (33.0)
ALT, U/L	50.1 (40.1)
ALP, U/L	286.6 (112.5)
γ-GTP, U/L	46.2 (45.1)
BUN, mg/dL	14.9 (3.7)
Cr, mg/dL	0.76 (0.20)
eGFR, mL/min/1.73	69.05 (14.42)
UA, mg/dL	5.56 (1.33)
TC, mg/dL	167.2 (30.2)
LDL, mg/dL	90.3 (26.3)
HDL, mg/dL	49.8 (14.1)
TG, mg/dL	118.5 (164.8)
Glu, mg/dL	106.0 (27.6)
HbA1c, %	5.95 (0.77)
AFP, ng/mL	16.91 (39.15)
DAA Regimens (ASV/SOF/OBV), No.	50/41/4

Abbreviations: AFP, alpha-fetoprotein; Alb, albumin; ALP, alkaline phosphatase; ALT, alanine aminotransferase; AST, aspartate aminotransferase; BMI, body mass index; BUN, blood urea nitrogen; Cr, creatinine; eGFR, estimated glomerular filtration rate; γ-GTP, gamma-glutamyltransferase; Glu, glucose; Hb, hemoglobin; HDL, high density lipoprotein; INR, international normalized ratio; LDL, low density lipoprotein; Plt, platelet; Tbil, total bilirubin; SD, standard deviation; TC, total cholesterol; TG, triglyceride; UA, uric acid; WBC, white blood cell. ^a^ Values are expressed as mean (SD) unless otherwise indicated.

**Table 2:** Independent Risk Factors for Zinc Deficiency Two Years after Sustained Virologic Response (Zn-2y).

Parameters	OR (95% CI)	Chi-Square Test *p* Value	OR (95% CI)	Multivariate Analysis *p* Value
Baseline Zn < 65 μg/dL	9.47 (2.73–32.86)	<0.001	10.56 (3.00–45.27)	<0.001
FIB-4 Index > 3.25	15.38 (1.94–122.13)	0.001		
Plt ≤ 120 × 10^3^/µL	5.17 (1.53–17.53)	0.005		
Alb ≤ 3.5 g/dL	10.21 (3.06–34.07)	<0.001		
INR > 1.1	7.08 (1.86–26.90)	0.001		
AST > 40 U/L	5.45 (1.44–20.63)	0.005		
UA > 5.5 mg/dL	8.80 (1.87–41.32)	0.001	9.99 (2.31–71.31)	0.001
TC ≤ 160 mg/dL	7.47 (1.96–28.42)	0.001		

Multivariate analysis revealed that baseline Zn < 65 μg/dL (OR = 10.56; 95% CI, 3.00–45.27; *p* < 0.001) and baseline UA > 5.5 mg/dL (OR = 9.99; 95% CI, 2.31–71.31; *p* = 0.001) were independent risk factors of zinc deficiency two years after SVR (Zn-2y < 70 µg/dL). Abbreviations: Alb, albumin; AST, aspartate aminotransferase; CI, confidence interval; INR, International Normalized Ratio; OR, odds ratio; Plt, platelets; TC, total cholesterol; UA, uric acid; Zn-2y, zinc level two years after sustained virologic response (SVR); Blank cells: parameters that are not independent factors.
